# Hepatitis E Virus Mixed Infection in Immunocompetent Patient

**DOI:** 10.3201/eid1903.121510

**Published:** 2013-03

**Authors:** Donald B. Smith, Jeff Vanek, Louise Wellington, Ingolfur Johannessen, Sandeep Ramalingam, Peter Simmonds

**Affiliations:** Author affiliations: University of Edinburgh, Edinburgh, Scotland, United Kingdom (D.B.S., P.S.);; Royal Infirmary of Edinburgh, Edinburgh (J.V., I.J., S.R.);; NHS Lothian, Edinburgh (L.W.)

**Keywords:** Hepatitis E virus, mixed infection, viruses, immunocompetent

## Abstract

We detected 2 hepatitis E virus (HEV) strains in an acutely infected immunocompetent patient. Two populations of genotype 3 virus were observed in the hypervariable regions and open reading frames 2 and 3, indicating multiple infection with hepatitis E virus. Persons with mixed infections may provide the opportunity for virus recombination.

Most reports of multiple infection with different hepatitis E virus (HEV) variants can be ascribed to immunodeficiency of the host or to high frequencies of infection. For example, 2 different genotype 3 subtypes were detected in samples from an immunocompromised kidney transplant recipient (*1*), and virus from another immunocompromised patient contained an insertion of host-derived sequences or various deletions of this sequence (*2*). Multiple infection or co-circulation of closely related virus variants is not unexpected in such patients with chronic HEV infection (references in [*3*]). Few cases of multiple infection of immunocompetent persons have been reported; 2 different virus genotypes were isolated from a sushi chef in Japan (*4*), and 2 persons in Nepal were each infected by different genotype 1 subtypes (*5*). In the course of our study of the open reading frame (ORF) 1 hypervariable region (HVR) variability in acutely infected persons in Scotland (*6*), we described a person infected with 2 variants of HEV that encoded dramatically distinct HVR sequences. We report here a more complete investigation of this case.

## The Study

The patient, a 55-year-old man, sought treatment at a hospital in the southeast of Scotland in April 2012 for nausea, vomiting, anorexia, jaundice, headache, and abdominal pain. At admission, the patient’s blood sample was positive for anti-HEV IgM and IgG and raised levels of alanine aminotransferase (ALT) (4,023 IU/L; reference range 5–60 IU/L) and bilirubin (91 μmol/L; reference range 3–17 μmol/L). Over the next 10 days, ALT declined steadily to 404 IU/L and returned to normal levels 1 month later. The patient’s abdominal pain had largely resolved by 6 weeks after seeking treatment, but fatigue and lethargy persisted for >8 weeks. There was no evidence that the patient was immunocompromised or was co-infected with other hepatitis viruses. The patient had unexplained jaundice at age 10 years and a history of alcohol abuse as an adult. Symptoms were relatively severe and required 6 days of hospitalization. Informed consent was provided by the patient for laboratory assessment of blood samples.

Virus RNA was extracted from 140 μL of serum and sequenced at limiting dilution in the HVR and ORF2 regions as described (*6*). Analysis of the ORF2/ORF3 overlap region used the primers 5′-CGGGTGGAATGAATAACATGT-3′ (outer sense nucleotides 5098–5118 relative to M73218), 5′-GCRGTYARCGGCGMRGCCCCAGCTG-3′ (outer antisense, 5481–5457), 5′-TYTGCCTATGCTGCCCGCGCCACCG-3′ (inner sense, 5184–5209) and 5′-GGCGCTGGGMYTGGTCRCGCCAAG-3′ (inner antisense, 5426–5403). Negative controls were included in all experiments. GenBank accession numbers for the ORF2 and ORF2/3 sequences described here are JX516004–JX516053 and for the HVR sequences are JX270882–JX270902.

Nucleotide sequence analysis of the HVR from individual virus genomes sampled by limiting dilution of cDNA revealed 2 distinct genotype 3 populations (populations A and B, Figure). Members of these populations all differed by the same 18 nucleotide substitutions, of which 13 were synonymous and 5 nonsynonymous. Two populations (A and B) were also observed among 25 ORF2 sequences (the 2 populations differed by 5 synonymous substitutions) and 26 ORF2/ORF3 sequences (the 2 populations differed by a single substitution, which was nonsynonymous in ORF2 but synonymous in ORF3). 

**Figure:**
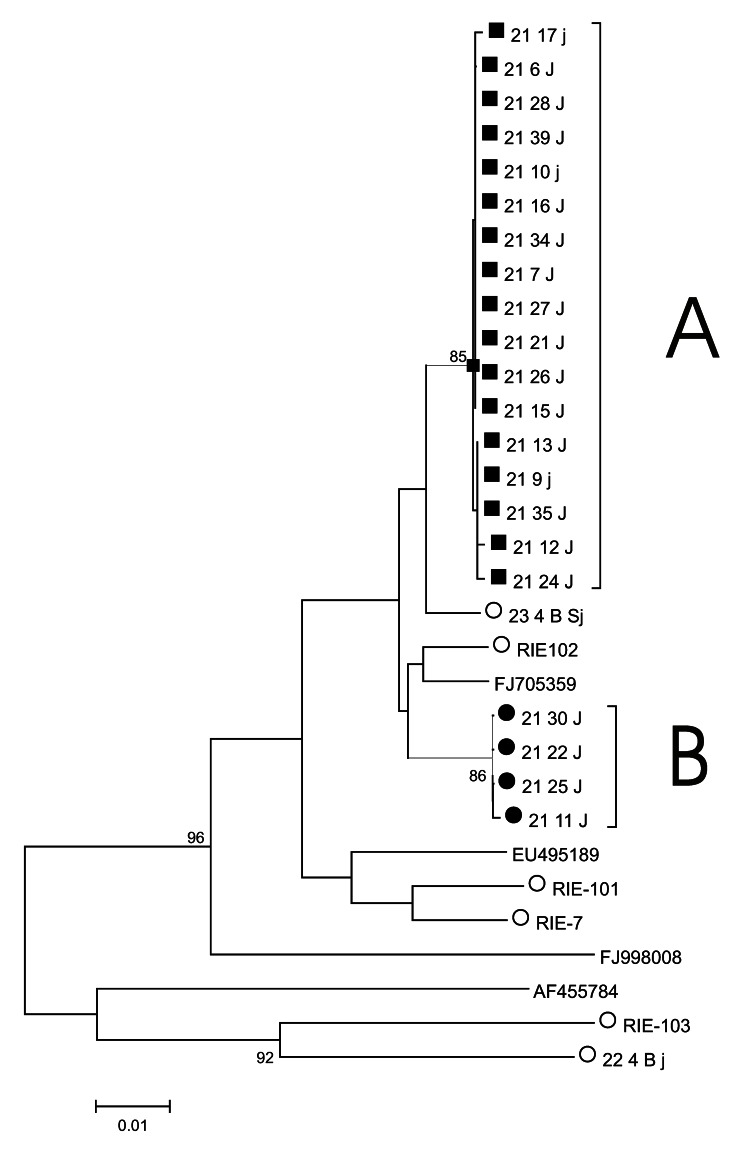
Phylogenetic tree of hepatitis E virus hypervariable region variants. A neighbor-joining tree of hypervariable region sequences was constructed by using MEGA 4 (*15*). A and B indicate sequences for virus populations A and B. Sequences from the patient studied here (patient 21) belonging to virus population A are indicated by a solid square, and those belonging to virus population B are indicated by a solid circle. Other sequences obtained in the same laboratory are indicated by an open circle. Closely related genotype 3 sequences obtained from GenBank are identified by their accession number. Bootstrap support (1,000 replicates) for branches of >70% are indicated. Scale bar indicates nucleotide substitutions per site.

In all 3 regions, 1 of the virus populations represented ≈80%–90% of the sequences: in HVR, 17 sequences were population A and 4 population B; in ORF2, 21 sequences were population A, 2 population B, and 1 mixed; and in ORF2/3, 20 sequences were population A, 4 population B, and 2 mixed. The 3 mixed sequences contained ambiguities at positions where the 2 populations differed, implying that templates from both populations were present in the limiting dilution reaction. The expected number of such mixed-template reactions can be calculated by using the Poisson distribution and the number of PCR-negative replicate reactions**,** which were 47% (HVR), 50% (ORF2), and 43% (ORF2/3). Thus, 17.5%, 15.4%, and 20.8%, respectively, of reactions would be expected to contain >1 template, of which, given the ratio of populations A and B in each region, 31%, 17.2%, and 28%, respectively, would contain templates from different populations, equivalent to 5.4%, 2.6%, and 5.8%, respectively, of positive reactions, compared with the 4.2% (3/71) observed. All other substitutions in these sequence sets were unique to a single sequence. A trivial explanation for the observation of mixed populations in this sample would be contamination during sample collection, processing**,** or analysis. However, this appears unlikely, given the low frequency of HEV infection in the United Kingdom, the lack of PCR products in negative controls, and the distinctness of the sequences obtained from those previously studied in our laboratory.

Infection of the patient in this study with 2 HEV variants could arise for several different reasons. First, the person could have been exposed to a homogeneous source of HEV, which then diversified during infection. Assuming that infection occurred 1 month before the first symptoms and 6 weeks before sampling, a divergence of HVR sequences at 18 (7.4%) of 243 nucleotide positions equates to a rate of nucleotide substitution from a presumed ancestor of both populations of 0.32/site/year. Not only were these substitutions mostly synonymous, and therefore unlikely to represent selection during infection, but the inferred rate of nucleotide substitution would be several orders of magnitude higher than those previously reported for HEV (0.0014/site/year) (*7*) or for the HCV HVR (0.0043/site/year) (*8*). In addition, the divergence between the 2 virus populations contrasts with the homogeneity observed among 7 other acutely infected patients and between viruses transmitted from 1 person or host to another (*6*).

A second possibility is that this person had a subclinical chronic HEV infection and then became superinfected with a second virus that induced jaundice. No previous blood samples were available for testing, but a prior subclinical chronic infection seems unlikely, given the absence of immunosuppression; the presence of anti-HEV IgM and IgG; the decline and increase, respectively, in these antibody titers in a sample collected 7 days later; and the decline in ALT and bilirubin levels in the weeks following the patient’s hospital visit.

Last, the clinical features and our immunologic and virologic findings are consistent with the patient having been infected from a single source containing >1 variant, such as pig-derived figatellu sausage (*9*). Alternatively, he might have been multiply infected from different sources within in a short period and before the development of protective immunity. Follow-up interview revealed no occupational exposure to animals, apart from a domestic dog, and no recent foreign travel; he spent 3 days in a beach resort in southeastern Scotland 12 days before onset of symptoms. The patient ate supermarket-bought prewashed salad vegetables and fresh fruit and drank only bottled or tap water. The patient ate fish, shellfish, chicken, pork, bacon, sausages, ham, Brussels paté, and eggs but not venison or pig liver. He regularly handled uncooked pork and beef at home while preparing food. HEV has been found on the hands and gloves of 17% of persons selling pork products (*10*).

## Conclusions

This study suggests that mixed infection with HEV can occur in immunocompetent persons with no obvious high-risk exposure to HEV-infected food or sewage. Only about 300 cases of acute HEV are reported each year in England and Wales (*11*), and the prevalence of HEV PCR-positive blood donors in England is 0.014% (*12*). However, the seroprevalence of anti-HEV IgG is 1,000× higher (16%) (*13*), and in this context, the identification of sources of autochthonous HEV infection remains an important goal. Our results are also relevant to the suggestion that HEV may undergo recombination (*14*). The case described here suggests that the conditions required for virus recombination may occasionally arise in immunocompetent persons.
